# *Bifidobacterium* mixture (*B longum* BB536*, B infantis* M-63*, B breve* M-16V) treatment in children with seasonal allergic rhinitis and intermittent asthma

**DOI:** 10.1186/s13052-017-0340-5

**Published:** 2017-03-07

**Authors:** Michele Miraglia Del Giudice, Cristiana Indolfi, Michele Capasso, Nunzia Maiello, Fabio Decimo, Giorgio Ciprandi

**Affiliations:** 1 Department of Woman, Child and General and Specialized Surgery, Second University of Naples, Naples, Italy; 2 Department of Paediatrics, Ospedale Civile ‘Ave Gratia Plena’, Piedimonte Matese, CE Italy; 3 Department of Medicine, IRCCS-AOU San Martino-IST, Genoa, Italy

**Keywords:** Bifidobacteria, Probiotic, Quality of life, Allergic rhinitis, Intermittent asthma, Children

## Abstract

**Background:**

Allergic rhinitis (AR) and allergic asthma are caused by an IgE-mediated inflammatory reaction. Probiotics may exert anti-inflammatory and immune-modulatory activity. Thus, this study aimed at investigating whether a Bifidobacteria mixture could be able to relieve nasal symptoms, and affect quality of life (QoL) in children with AR and intermittent asthma due to *Parietaria* allergy.

**Materials and methods:**

The present study was conducted as placebo-controlled, double-blinded, and randomized. Globally, 40 children (18 males; mean age 9 ± 2.2 years) were enrolled. They were treated with probiotics or placebo: 1 sachet/day for 4 weeks. AR symptoms, and QoL were assessed at baseline and after treatment. Use of rescue medications, such as cetirizine syrup and salbutamol spray, was also permitted and recorded.

**Results:**

Children treated with probiotic mixture achieved a significant improvement of symptoms (*p* < 0.005), and QoL ((*p* < 0.001). Placebo group had worsening of symptoms (*p* < 0.005) and QoL (*p* < 0.001). The use of rescue medications was overlapping in the two groups. The intergroup analysis showed that probiotic mixture was significantly superior than placebo for all parameters.

**Conclusions:**

The current study demonstrated that a Bifidobacteria mixture was able of significantly improving AR symptoms and QoL in children with pollen-induced AR and intermittent asthma.

**Clinical trial registration:**

ClinicalTrials.gov ID NCT02807064.

## Background

Seasonal allergic rhinitis (SAR) is common in children and is characterized by typical symptoms and nasal inflammation after pollen exposure [1, 2]. Anyhow, allergic asthma is characterized by airway inflammation and bronchial hyperreactivity; asthma symptoms are also triggered by allergen inhalation [3]. Actually, allergy is defined by a functional defect of allergen-specific T regulatory cells allowing T helper 2 (Th2) cells polarization [4]. Th2 cells produce interleukins, including IL-4, IL-5, and IL-13, that drive allergic inflammation. Moreover, allergic rhinitis affects quality of life (QoL) in children [5], and is the main risk factor for asthma onset and worsening and is a common co-morbidity in asthmatics [6]. Patients with Parietaria allergy may present AR associated with asthma.

Allergy prevalence has dramatically worldwide increased over the past decades and the hygiene hypothesis suggested that reduced exposure to microbial agents could increase the risk of allergic diseases [7]. This theory has been recently revisited emphasizing the role of microbiota dysbiosis promoting impaired immunological tolerance to allergens [8].

The main medications for allergy are antihistamines, bronchodilators, and corticosteroids, which are effective, but may have adverse events. For this reason, many people prefer to use also non-pharmacological medicine. In this regard, increasing attention has been paid to probiotics. Probiotic is a life microorganism able to give healthy effects on the host [9]. Probiotics may exert many immune-modulatory and anti-inflammatory effects [10, 11]. So, many attempts of restoring enteric microbiota with probiotic supplementation were performed in allergic disorders [12, 13]. In this regard, *Lactobacilli* were able to reduce allergic symptoms and improve QoL also in children [14]. There is evidence that probiotics promote the production of some cytokines, including IL-10, TGF-β, IL-12, and IFN-γ, that regulate the immune response and damp allergic inflammation [15–19].

Bifidobacteria were also extensively evaluated. *B. breve* reduced negative effects induced by antibiotics and restored enteric microbiota [20–23]; it also improved symptoms of atopic dermatitis [24]. *B longum* BB536 is widely used in dairy industry because of its anti-infective activity [25]. *B longum* BB536 treatment in SAR patients significantly reduced nasal symptoms and Th2-polarized immune response [26]. *B infantis* M-63 is commonly used in formula. However, Bifidobacteria colonization depends on age of subjects and specific strain [27, 28].

Recently, it has been demonstrated that a Bifidobacteria mixture, containing *B longum* BB536 (3x10^9^ CFU), *B infantis* M-63 (1x10^9^ CFU), and *B breve* M-16 V (1x10^9^ CFU), has several biological characteristics, including in vitro compatibility, stability over time, and optimal doses, able to suggest its clinical use [29]. So, this mixture has been marketed as medical device I class. Therefore, the aim of the current randomized, double-blind, placebo-controlled study was to evaluate the effects of this Bifidobacteria mixture in children with SAR and intermittent asthma. The primary outcome was the effect on AR symptoms, secondary outcome was effects on QoL.

## Methods

The present study was conducted as prospective, double-blind, placebo-controlled, and randomized. Globally, 40 children (18 males; mean age 9 ± 2.2 years) were enrolled. They attended the Department of Woman, Child and General and Specialized Surgery of the Second University of Naples, as suffering from SAR and well controlled asthma. AR and asthma diagnosis were performed, according to GINA and criteria [3, 30]. In particular, allergy was defined if symptom history was consistent with documented sensitization, such as positive skin prick test, to *Parietaria* allergen, i.e. allergic symptoms had to occur only during the Parietaria pollen season. AR diagnosis was done on the basis of typical symptoms occurring during Parietaria pollen season. Asthma diagnosis was performed considering typical symptoms and functional assessment.

Inclusion criteria were: i) age range between 4–17 years, ii) diagnosis of SAR and intermittent asthma due to *Parietaria officinalis* pollen, iii) presence of AR symptoms, including nasal itching, sneezing, watery rhinorrhea, nasal obstruction, and itchy eyes, since at least two weeks, documented by a run-in period, iv) well controlled asthma, such as symptoms requiring short acting β2-agonist ≤ 2 times/month, and v) written informed consent signed by parents. Exclusion criteria were: i) concomitant respiratory infections, ii) chronic illnesses, iii) continuous use of medications (antihistamines and corticosteroids) in the last 2 weeks, and iv) not well controlled asthma.

Patients were randomly (1:1 ratio) subdivided in two groups: placebo-treated (Group A) and actively-treated (Group B). Active medication was an oral supplementation containing Bifidobacteria mixture, *B longum* BB536 (3x10^9^ CFU), *B infantis* M-63 (1x10^9^ CFU), and *B breve* M-16 V (1x10^9^ CFU) as powder in 3 mg sachet. Placebo was an inert excipient as powder. Patients were instructed to assume 1 sachet diluted in little tepid water or milk in the morning for 8 weeks. Cetirizine syrup (1 drop/3Kg/bw) and salbutamol spray (200 mcg/puff) were permitted as rescue medication during both run-in and treatment periods; the symptomatic use was recorded on a diary card. About cetirizine, it was calculated the number of days when it was assumed. About salbutamol, the number of puff was considered.

Adverse events were registered. The study was performed during the *Parietaria* pollen season. The study protocol was approved by the Ethics Committee of the Second University of Naples. The study was registered at ClinicalTrials.gov ID NCT02807064.

The study was conducted during the 2015 spring.

Children were evaluated at baseline (T0) and at the end of the treatment (T1).

Nasal symptoms (nasal itching, sneezing, rhinorrhea, nasal obstruction, and itchy eyes) were scored using a four-point scale (0 = no symptom; 1 = mild; 2 = moderate; 3 = severe) and the sum was calculated as total symptom score (TSS).

QoL was assessed by the Mini Rhinoconjunctivitis quality of life questionnaire [31].

### Lung function was assessed by spirometry at baseline

The sample size was calculated by log-rank test with power at 90% and α error at 5%: 20 subjects per arm were considered sufficient. Randomization was performed per blocks following the Wichmann-Hill model.

Data were reported as medians and interquartile ranges (IQR) and standard deviation. The Mann–Whitney test was used as a non-parametric counter-part, paired data were compared using the Wilcoxon test. A *P*-value less than 0.05 was considered statistically significant. A statistical software program (StatSoft Italia s.r.l. 2005. Statistica, Vigonza, Italy) was used for all the analyses.

## Results

All children completed the study; both treatments were well tolerated and there were no clinically relevant side effects in children of both groups. The adherence to treatments was >90% in both groups.

There was no significant difference between groups at baseline, including baseline lung function (normal in all subjects) so the two groups were homogeneous.

### Symptoms

**Fig. 1:**
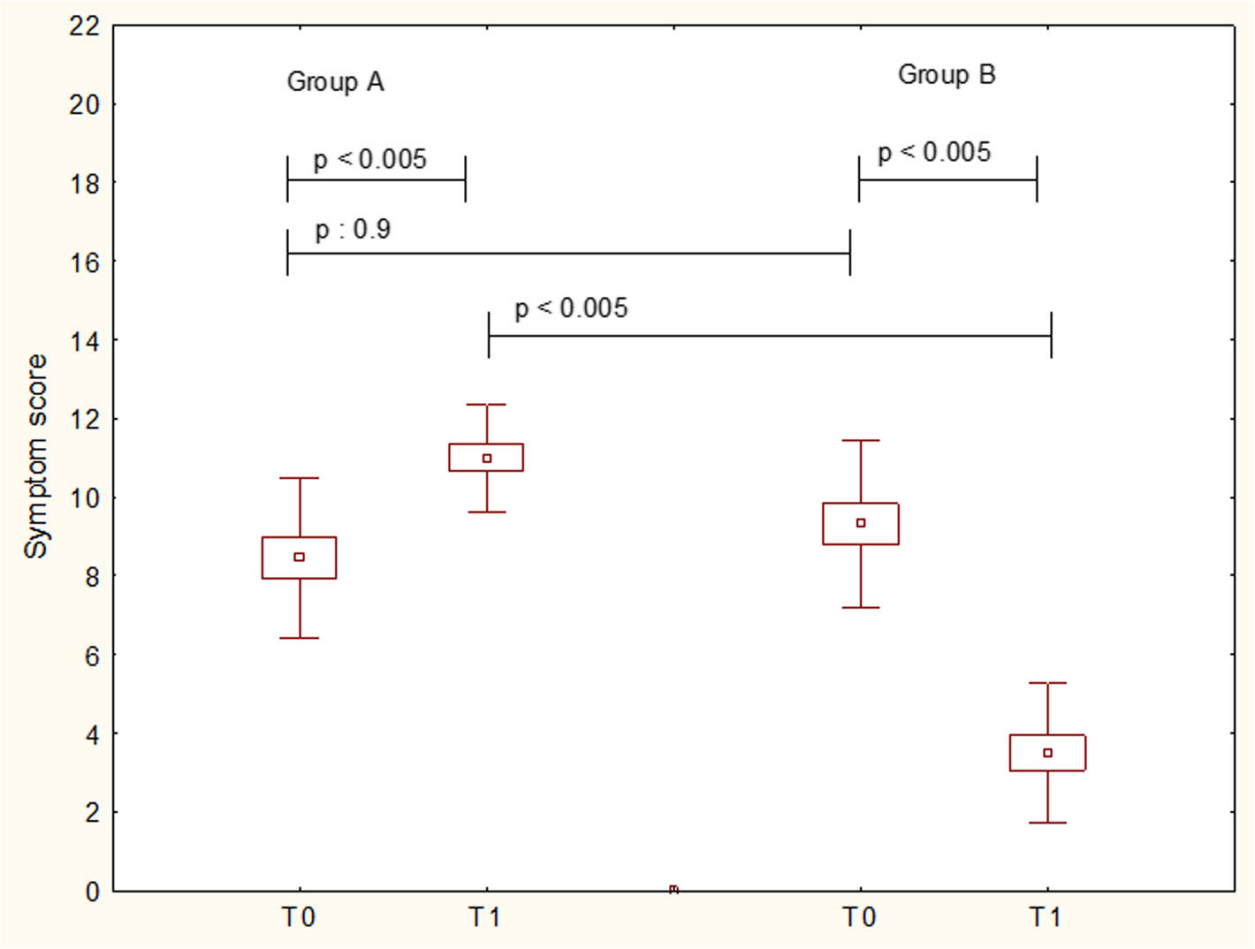
Total symptom scores in patients treated with Placebo (Group A) or Bifidobacteria mixture (Group B) at baseline (T0) and after treatement (T1). Data are expressed as medians, IQR, and standard deviations

TSS significantly (*p* < 0.005) increased in Group A (T0 = 8.4 ± 2; T1 = 11 ± 1.3); whereas significantly decreased in Group B (T0 = 9.3 ± 2.1; T1 = 3.5 ± 1.7), as reported in Fig. 1. The intergroup comparison showed that there was a significant difference (*p* < 0.005) at T1.

### Quality of life

**Fig. 2:**
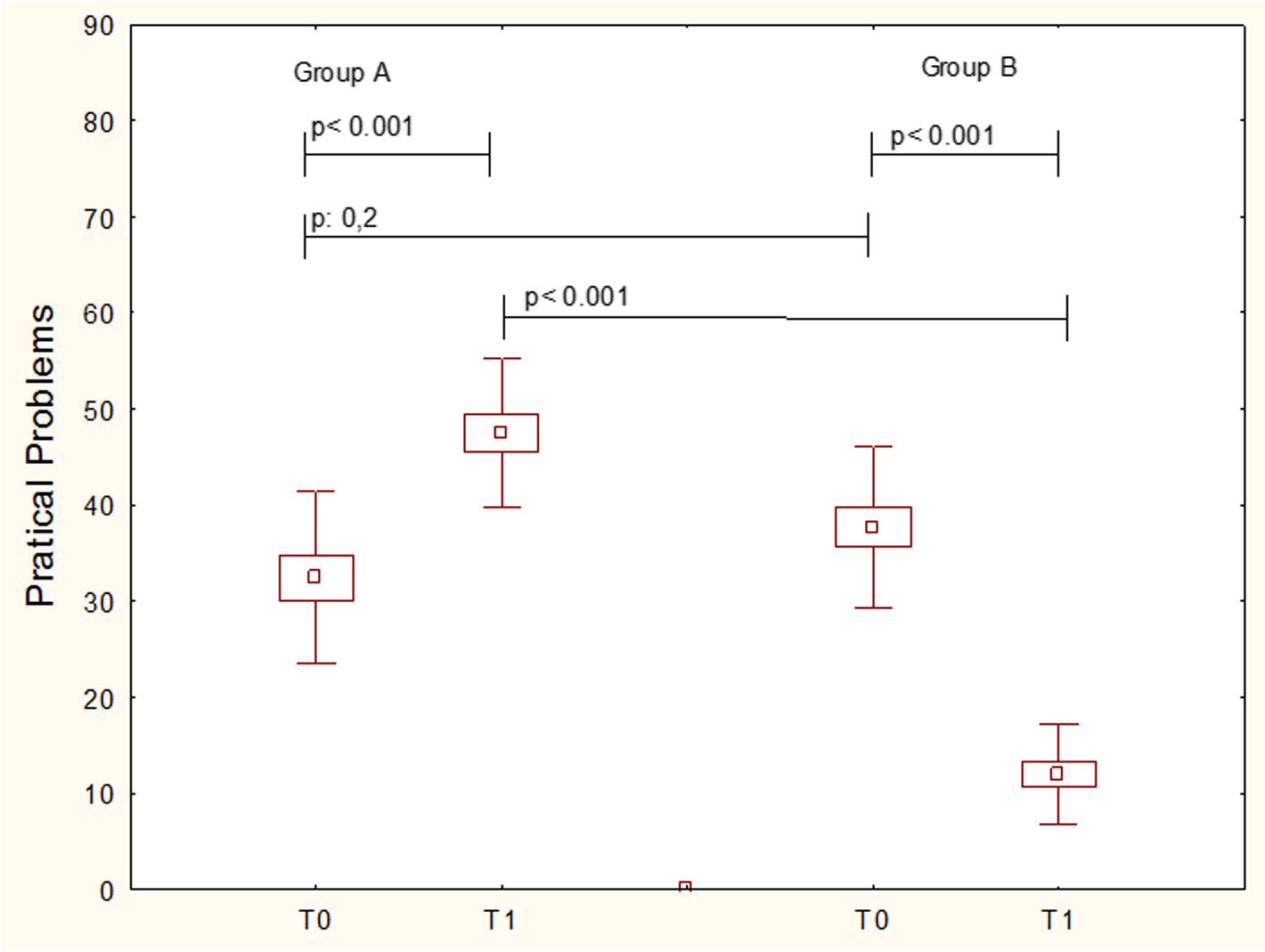
QoL = practical problems item in patients treated with Placebo (Group A) or Bifidobacteria mixture (Group B) at baseline (T0) and after treatement (T1). Data are expressed as medians, IQR, and standard deviations

The only item that significantly changed in both groups during the study was the practical problems. It significantly (*p* < 0.001) increased in Group A (T0 = 32.4 ± 8.9; T1 = 47.5 ± 7.1); whereas significantly (*p* < 0.001) decreased in Group B (T0 = 37.6 ± 8.4; T1 = 11.9 ± 5.2), as reported in Fig. 2. The intergroup comparison showed that there was a significant difference (*p* < 0.001) at T1.

### Rescue medication

Cetirizine use was overlapping in two groups (33 ± 7 days in Group A and 31 ± 8 days in Group B). Salbutamol use was also overlapping in the two groups (22 ± 4 puffs in Group A and 19 ± 7 puffs in Group B).

## Discussion

The rationale for using probiotics in allergic disorders is robust and is based on the concept that the balance between immunologic tolerance and inflammation is regulated by crosstalk between intestinal microbiota and innate and adaptive immune response [32]. In particular, the main targets of probiotics are: dendritic cells, epithelial cells, T regulatory cells, effector lymphocytes, natural killer cells, and B cells. Probiotics modulate allergic response mainly by stimulating Th1 pathway and restoring T regs. So, probiotics may effectively modulate the impaired immune response in allergic subjects.

*Bifidobacteria* are Gram-positive anaerobic bacteria and are ubiquitous and endosymbiotic inhabitant of the digestive tract. As they have beneficial effects on human health, are commonly used in the production of probiotics products. However, probiotics to be fruitfully and safely used should be respond to a series of requisites. In this regard, it has been performed an in vitro study to assess the use of *B longum* BB536, *B infantis* M-63, and *B breve* M-16 V in combination as food supplement [29]. Growth compatibility, resistance to antimicrobial agents, resistance to gastric acidity, bile salt hydrolysis, and adhesion to the human epithelial cells were examined. This study showed that these strains had reliable characteristics confirming their probiotic belonging. A further in vitro study examined the effects of this Bifidobacteria mixture on dendritic cells functionality from children with inflammatory bowel disease [33]. It was demonstrated that Bifodobacteria improved the antigen uptake and processing exerted by dendritic cells from children with Crohn’s disease, but there was no relevant effect in children with ulcerative colitis or functional gastrointestinal disorders. The authors concluded that this immunological effect could damp the impairment of intestinal innate immunity and reduce uncontrolled microbial overgrowth. An in vivo confirmatory study has been recently published [34]. This study explored the possibility that the Bifidobacteria mixture could improve abdominal pain and quality of life in children with irritable bowel syndrome and functional dyspepsia. Actually, this compound achieved the expected outcomes.

On the basis of this background, we tested the hypothesis that this Bifidobacteria mixture could positively affect AR symptoms and QoL in children with SAR and intermittent and well-controlled asthma. Really, the present study showed that a *Bifidobacteria* mixture was significantly effective in reducing nasal symptoms and in improving QoL in children with SAR and intermittent asthma.

A possible explanation of these findings could be dependent on the modulatory activity on innate immunity. It seems plausible that Bifidobacteria may orient the immune response toward a physiological Th1-polarized immune response and may restore a Treg pathway able to reducing allergic inflammation. Consequently, symptoms diminished.

Probiotics may be able to affect immune response and consequently allergic disorders. This matter is up-to-date in the prevention and treatment of respiratory disorders [12]. Two recent meta-analysis pointed out the role of probiotics in the management of allergic rhinitis and prevention of upper respiratory infection [13, 35].

It is to note that the effectiveness of probiotics is usually specific for each single strain. In this regard, there is a body of evidence that *Lactobacillus acidophilus* NCFM and *Bifidobacterium lactis* BL-04 exert different anti-inflammatory effects and modulate allergic reaction, diminishing the Th2-polarization, promoting Th1 proliferation, and restoring the defective Treg function [36–38]. Therefore, these strains have been fruitfully used in the treatment of pollen-induced allergic rhinitis [39]. Moreover, probiotics may be also effective in the treatment of food allergy [40] as well as gastric infection [41].

So, our findings are substantially consistent with these previous reports.

Noteworthy, the current compound significantly improved QoL item concerning the practical problems strictly closed to nasal symptom severity. Thus, this outcome underlines the effectiveness of this compound. However, a placebo effect could be not excluded a priori*.*

On the other hand, the main limitation of this study is the limited number of enrolled patients. So, it should be considered as preliminary experience that should be possibly confirmed by a multicenter trial enrolling larger population of allergic children.

## Conclusion

The current study demonstrated that a *Bifidobacteria* mixture was able of significantly improving AR symptoms and QoL in children with pollen-induced AR and intermittent asthma.
